# Alterations in the Blood Kynurenine Pathway Following Long-Term PM2.5 and PM10 Exposure: A Cross-Sectional Study

**DOI:** 10.3390/biomedicines12091947

**Published:** 2024-08-26

**Authors:** Churdsak Jaikang, Giatgong Konguthaithip, Yutti Amornlertwatana, Narongchai Autsavapromporn, Sirichet Rattanachitthawat, Tawachai Monum

**Affiliations:** 1 Department of Forensic Medicine, Faculty of Medicine, Chiang Mai University, Chiang Mai 50200, Thailand; churdsak.j@cmu.ac.th (C.J.); giatgong_k@cmu.ac.th (G.K.); yutti.amornlert@cmu.ac.th (Y.A.); 2 Metabolomics Research Group for Forensic Medicine and Toxicology, Department of Forensic Medicine, Faculty of Medicine, Chiang Mai University, Chiang Mai 50200, Thailand; 3 Division of Radiation Oncology, Department of Radiology, Faculty of Medicine, Chiang Mai University, Chiang Mai 50200, Thailand; narongchai.a@cmu.ac.th; 4 General Education Office, Faculty Agricultural Technology, Burapha University, Sakaeo 27160, Thailand; sirichet@buu.ac.th

**Keywords:** PM2.5, kynurenine pathway, ^1^-NMR spectroscopy, tryptophan, particulate matters

## Abstract

Human exposure to PM2.5 and PM10 has been linked to respiratory and cardiovascular diseases through inflammation activation. The kynurenine pathway is associated with inflammation, and it is necessary to investigate the effects of long-term PM2.5 and PM10 exposure on this pathway. This study aimed to conduct a cross-sectional analysis of long-term PM2.5 and PM10 exposure’s impact on the kynurenine pathway using proton NMR spectroscopy (^1^H-NMR). The participants were divided into a low-PM-exposure group (LG; *n* = 98), and a high-PM-exposure group (HG; *n* = 92). The metabolites of tryptophan were determined in blood by ^1^H-NMR. Serotonin, cinnabarinic acid, xanthurenic acid, 5-hydroxytryptophan, indoleacetic acid, tryptamine, melatonin, L-tryptophan, 5-hydroxy-L-tryptophol, indoxyl, 2-aminobenzoic acid, 5-HTOL, hydroxykynurenine, L-3-hydroxykynurenine, *N*-formyl kynurenine, 3-hydroxy anthranilic acid, kynurenic acid, and picolinic acid significantly increased (*p* < 0.05) in the HG group. Conversely, NAD and quinolinic acid significantly decreased in the HG group compared to the LG group. The enzyme activities of indoleamine 2,3-dioxygenase and formamidase significantly decreased, while kynureninase and kynurenine monooxygenase significantly increased. The kynurenine pathway is linked to inflammation and non-communicable diseases. Disruption of the kynurenine pathway from particulate matter might promote diseases. Reducing exposure to the particulate matter is crucial for preventing adverse health effects.

## 1. Introduction

Air pollution is one of the environmental problems affecting public health worldwide. Air pollution, especially in Northern Thailand, is the most significant environmental issue because forest fires and the burning of agricultural land are the primary sources of air pollution in this area [1,2,3]. Farmers clean their agricultural waste products annually by burning them during the dry season. High levels of particles less than 2.5 µm in diameter (PM2.5) and particles less than 10 µm in diameter (PM10) have been reported in Northern Thailand during the haze period [1,4,5]. High levels of PM2.5 and PM10 are serious air pollution problems worldwide that have been linked to adverse effects on human health and pose significant health risks to the human population [6,7].

PM2.5 and PM10 exposure is associated with health impacts on many human systems (including respiratory [8], cardiovascular [9], neurological [10], and reproductive systems), as well as being associated with developmental effects and increased mortality rates [11]. Toxic compounds, particularly polycyclic aromatic hydrocarbons (PAHs), are formed during incomplete combustion of organic matter and can be emitted from forest fires [12]. The aryl hydrocarbon receptor (AHR) is a latent transcription factor with numerous roles in generating oxidative stress [13]. AHR can bind to and be activated by PM-associated PAHs in various test systems [14]. PAHs and associated environmental AHR ligands can cause a range of detrimental health impacts, including atherosclerosis, inflammatory diseases, cancer, and aging [14].

The kynurenine pathway is a fundamental metabolic pathway involved in degrading the essential amino acid tryptophan [15]. Kynurenine activation via AHR has activated the indoleamine 2,3-dioxygenase (IDO), the initial and rate-limiting step of the kynurenine pathway. *N*-formylkynurenine is then converted to kynurenine, which serves as the central metabolite of the pathway [16]. Kynurenine is further metabolized into several downstream metabolites, including kynurenic acid, 3-hydroxykynurenine, 3-hydroxyanthranilic acid, quinolinic acid, and picolinic acid. This pathway is central to several physiological processes, including the regulation of immune responses, the maintenance of neuronal function, and the synthesis of important molecules such as nicotinamide adenine dinucleotide (NAD+) [17].

The kynurenine pathway regulates tryptophan levels, which are crucial for protein synthesis and the creation of other biologically active compounds. Moreover, the kynurenine pathway is a critical pathway for synthesizing NAD+, which is vital for energy production and DNA repair. Metabolites from this pathway, such as kynurenic acid, contribute to neurotransmitter regulation [18,19,20], immune system modulation [21], and antioxidant protection [22]. Some of these metabolites, particularly kynurenic acid, shield against neurodegenerative disorders. Furthermore, the pathway exhibits both anti-inflammatory and pro-inflammatory properties, depending on the specific metabolites involved. The balance of these metabolites is crucial for maintaining normal physiological function and health. The kynurenine pathway and its metabolites are biomarkers in various physiological and pathological conditions, offering valuable insights into disease progression, treatment responses, and overall health status [23,24]. However, there is limited data for describing the effect of PM2.5 and PM10 exposure on the kynurenine pathway. Therefore, the aim of this study was to investigate the long-term effects of PM2.5 and PM10 exposure on the kynurenine pathway using proton nuclear magnetic resonance spectroscopy (^1^H-NMR).

## 2. Materials and Methods

### 2.1. Chemical and Reagents

Methanol, deuterium oxide (D_2_O) and 3-(trimethylsilyl)-[2,2,3,3-d4]-1-propionate sodium salt (TSP) were purchased from Sigma Aldrich (Saint Louis, MO, USA).

### 2.2. Sample Collection

This study was approved by the Ethics Committee, Faculty of Medicine, Chiang Mai University (RAD-2564-08613, Research ID: 8613). The volunteers were recruited in the Mae Jam District, Chiang Mai Province, for the high-exposure area and Watthana Nakhon District, Sa Kaeo Province, Thailand, for the low-exposure area. The average levels of PM2.5 and PM10 at high exposure ranged from 23–36 and 32–59 µg/m^3^/day, respectively. For the low-exposure area, the levels of PM2.5 and PM10 showed 17–25 and 9–45 µg/m^3^/day, respectively. One hundred and ninety (*n* = 190) healthy volunteers from each high- and low-PM2.5-exposure area were enrolled. Blood and urine samples were collected in two periods: before (October 2022) and during high levels of PM2.5 in ambient air (April 2023). Approximately 5 mL of blood samples were collected and contained in heparin tubes. Twenty mL of urine samples were contained in a plastic bottle. The samples were stored at −80 °C before analysis.

### 2.3. Preparation of Blood Samples

Blood samples were extracted with acetonitrile in a 1:1 ratio and then centrifuged at 4000 rpm at 4 °C for 10 min [25]. The supernatant was collected and lyophilized. The samples were mixed with 0.6 mL of deuterium oxide (D_2_O) containing 1 µM TSP and transferred to NMR tubes for analysis. The metabolite levels were measured using NMR at 500 MHz, employing a technique to suppress water resonance.

### 2.4. Acquisition Parameters

The proton NMR spectrum was recorded using a Bruker AVANCE 500 MHz instrument (Bruker, Bremen, Germany), equipped with a Carr-Purcell-Meiboom-Gill (CPMG, -RD-90°-(t-180°-t) n-acquire) pulse sequence for ^1^H-NMR measurements. The spectra were acquired at 27°C with water suppression pre-saturation. The acquisition parameters included 16 scans, a 1 s relaxation delay, a 3.95 s acquisition time, an 8278.146 Hz spectral window, a 0.126 Hz free induction decay (FID) resolution, and a 60.40 μs dwell time (DW). A 90° pulse with 16 signal averages (NSA) was applied. Baseline and phase corrections were performed using TopSpin 4.0.7 software. Spectra from 0 to 12 ppm were analyzed, with data normalized to the total integrated area. Metabolite resonances were identified using human databases.

### 2.5. Internal Standard

The 3-(trimethylsilyl)-[2,2,3,3-d4]-1-propionate sodium salt (TSP) was chosen as the internal standard. TSP is ideal for this application because all 14 protons within TSP share the same chemical environment, ensuring that the signal appears at a singular position at 0 ppm and 500 MHz. This signal arises from a region with a greater magnetic field intensity than other protons. Additionally, TSP is non-reactive in organic compounds and has a low boiling point, making it convenient to extract from the sample.

### 2.6. Peak Assignment and Chemical Identification

Each chemical compound was identified using the Human Metabolome Database (HMDB), retrieved in February 2024. Peak acquisition and J-coupling analysis were performed using the Bruker TopSpin version 4.0.7 software. The interpretation of the NMR spectra relied on the utilization of chemical shift values. These values played a key role in identifying the location of the signal for integration, determining the integrated area beneath the signal, analyzing spin-spin coupling, examining signal patterns, and evaluating the coupling constant. Each peak of every non-targeted metabolite needed to be identified and adjusted by less than 0.01 ppm compared to the HMDB database [26].

### 2.7. Data Analysis

The data were imported into MNOVA Software (version 12.0.0, MestreLab Research, Spain) for metabolite peak identification. All spectra in the chromatogram were baseline-adjusted to 0 and calibrated using the internal standard’s TSP peak at 0.000 ppm. After peak collection, the intensity of all metabolite peaks was calculated to concentration via the equation [27] shown below.

(1) $$\frac{I_{A}}{I_{B}}=\frac{H_{A}}{H_{B}}\;\times \;\frac{C_{A}}{C_{B}}$$

Where I = Signal intensity (Integral), H = Number of protons in a functional group, C = Concentration.

### 2.8. Metabolomics Measurements

MetaboAnalyst free online software (version 6.0) (http://www.metaboanalyst.ca/MetaboAnalyst, accessed on 26 July 2024) was employed to identify metabolomics data. Before data analysis, two classes, non-negative numbers for the compound concentration or peak intensity values, and missing value imputations were checked. Data were normalized by median, log-transformed (base 10), and auto-scaled for data scaling. Principal component analysis (PCA) and partial-least square discriminant analysis (PLS-DA) were performed to understand metabolite differences between the HG and LG groups. The Importance measure was analyzed using the variable importance in projection (VIP) and component. Quantitative enrichment analysis was used to predict disease signatures from blood samples. The study employed ROC curve analysis to evaluate the discriminatory capacities of the detected differential metabolites, and the area under the curve (AUC) was calculated.

### 2.9. Particulate Matter Level in Atmosphere and Average Daily Dose Exposure

PM2.5 and PM10 data were obtained from the Thai Meteorological Department between June 2014 and December 2023, and the levels of PM2.5 and PM10 were averaged. A year was assumed to consist of only 365 days to count lagged days in an integer format in µg/m^3^/day. The PM2.5 dose exposure was the average daily dosage (ADD) and was calculated as the following equation:

ADD = (C × IR × ET × ED)/(BW × AT)(2)

Here, C is the average value of PM2.5 or PM10 concentration by the Thai Meteorological Department between June 2014 and December 2023 (µg/m^3^). IR is the inhalation rate (m^3^/day), which for males is 17, and females 12. EF, ED, BW, and AT stand for exposure frequency (day/year), exposure duration (years), body weight (kg), and average exposure time (days).

### 2.10. Statistical Analysis

The results are shown in means ± standard deviation (SD). Normal distribution was tested by the Kolmogorov–Smirnov test. A comparison of high- and low-exposure groups was conducted by the Mann–Whitney U-test. The interrelationships between the time spent living in the area, the concentrations of PM2.5 and PM10, and the kynurenine metabolites were examined using Spearman’s rank correlation. The results with *p* < 0.05 were considered statistically significant.

## 3. Results

### 3.1. Demographic Data

One hundred and ninety participants were divided into two groups: the high-exposure group (HG) (*n* = 92) and the low-exposure group (LG) (*n* = 98). Gender, age, weight, height, blood pressure, and heart rate were similar between the groups. The results are presented in Table 1.

### 3.2. Determination of Metabolites in Blood Samples by ^1^H-NMR

**Figure 1:**
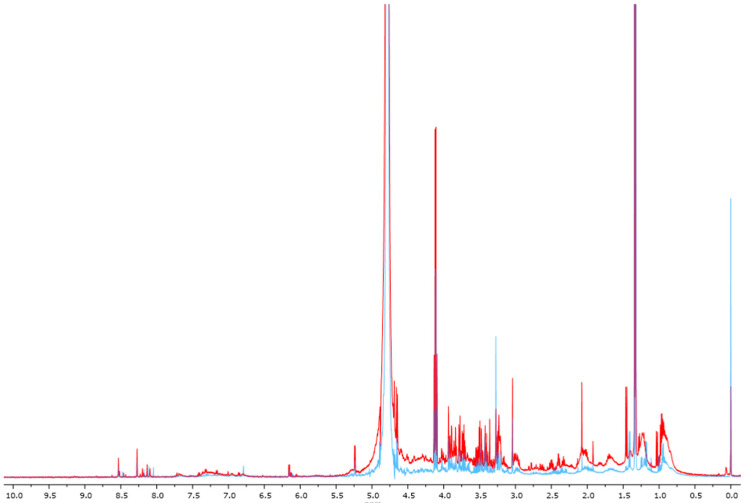
^1^H-NMR chromatogram of the blood samples of the high-PM2.5-exposure group (red) and low-PM2.5-exposure group (blue). When TSP = 0.00, serotonin = 3.10, cinnabarinic acid = 6.74, xanthurenic acid = 6.80, 5-HIAA = 6.81, 5-hydroxytryptophan = 6.82, indoleacetic acid = 7.15, tryptamine = 7.20, melatonin = 7.21, L-tryptophan = 7.28, 5-hydroxy-L-tryptophan = 7.29, indoxyl = 7.30, 2-aminobenzoic acid = 7.36, L-kynurenine = 7.37, 5-HTOL = 7.43, hydroxykynurenine = 7.48, L-3-hydroxykynurenine = 7.49, *N*-formyl kynurenine = 7.50, NAD = 7.60, 3-hydroxy anthranilic acid = 7.68, quinolinic acid = 7.89, kynurenic acid = 7.94 and picolinic acid = 7.95 ppm, respectively.

A 500 MHz ^1^H-NMR chromatogram of a blood sample is illustrated in Figure 1. The red and blue represent HG and LG, respectively. The tryptophan and its metabolites, including serotonin, cinnabarinic acid, xanthurenic acid, 5-HIAA, 5-hydroxytryptophan, indoleacetic acid, tryptamine, melatonin, L-tryptophan, 5-hydroxy-L-tryptophan, indoxyl, 2-aminobenzoic acid, L-kynurenine, 5-HTOL, hydroxykynurenine, L-3-hydroxykynurenine, *N*-formyl kynurenine, NAD, 3-hydroxy anthranilic acid, quinolinic acid, kynurenic acid, and picolinic acid were identified in the blood samples in both the HG and LG groups. The chemical shift of tryptophan and its metabolites is shown in the range 3.10–7.95. The chemical shifts of TSP, serotonin, cinnabarinic acid, xanthurenic acid, 5-HIAA, 5-hydroxytryptophan, indoleacetic acid, tryptamine, melatonin, L-tryptophan, 5-hydroxy-L-tryptophan, indoxyl, 2-aminobenzoic acid, L-kynurenine, 5-HTOL, hydroxykynurenine, L-3-hydroxykynurenine, *N*-formyl kynurenine, NAD, 3-hydroxy anthranilic acid, quinolinic acid, kynurenic acid, and picolinic acid were 0.00, 3.10, 6.74, 6.80, 6.81, 6.82, 7.15, 7.20, 7.21, 7.28, 7.29, 7.30, 7.36, 7.37, 7.43, 7.48, 7.49, 7.50, 7.60, 7.68, 7.89, 7.94 and 7.95 ppm, respectively.

### 3.3. Effect of PM2.5 Exposure on the Kynurenine Pathway

The tryptophan and its metabolism related to the kynurenine pathway were determined and compared between the groups. The results showed that serotonin, cinnabarinic acid, xanthurenic acid, 5-hydroxytryptophan, indoleacetic acid, tryptomine, melatonin, L-tryptophan, 5-hydroxy-L-tryptophol, indoxyl, 2-aminobenzoic acid, 5-HTOL, hydroxykynurenine, L-3-hydroxykynurenine, *N*-formyl kynurenine, 3-hydroxy anthranilic acid, kynurenic acid, and picolinic acid significantly increased in the HG group (*p* < 0.05), while NAD and quinolinic acid significantly decreased (*p* < 0.05) in the HG group compared with the LG group. The metabolite ratios of *N*-formyl kynurenine/L-tryptophan, L-kynurenine/*N*-formyl kynurenine, Kynurenic acid/L-kynurenine, 2-Aminobenzoic acid/L-kynurenine and L-3-hydroxykynurenine/L-kynurenine represent indolamine 2,3-dioxygenase (IDO), formamidase, kynurenine aminotransferase (KAT), kynureninase and kynurenine mono oxygenae (KMO), respectively. The IDO and formamidase activity significantly decreased, but kynureninase and KMO significantly increased (*p* < 0.05). The data are shown in Table 2.

### 3.4. Metabolomic Study

**Figure 2:**
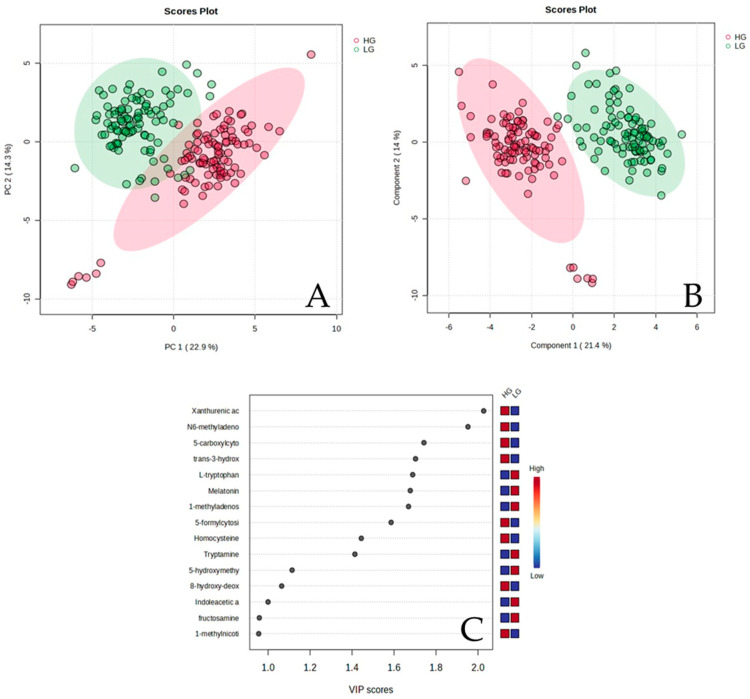
(**A**) Principal component analysis (PCA), (**B**) Partial least square-discriminant analysis (PLS-DA) of the high-(HG)-compared to the low-(LG)-exposure area to PM2.5 and PM10. (**C**) Top kynurenine metabolites detected via PLS-DA ranked based on variable importance projection (VIP) scores.

**Figure 3:**
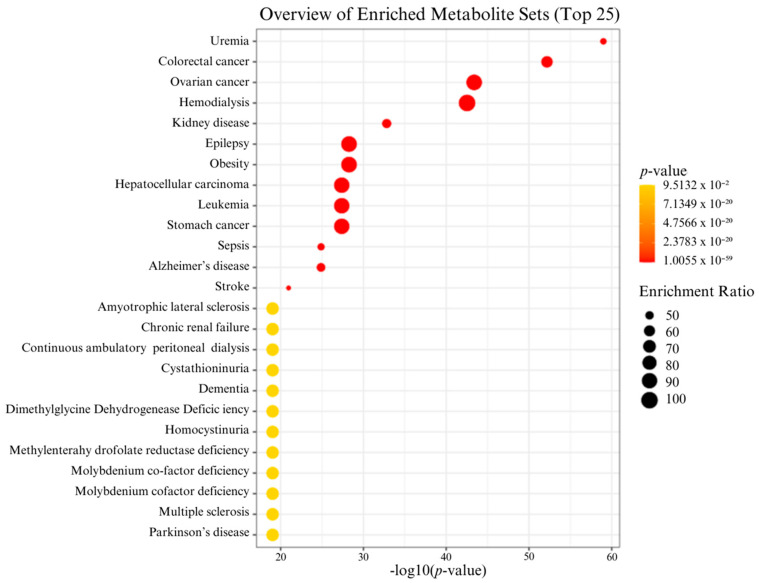
Overview of enriched metabolites sets related to kynurenine pathway disruption after long-term particulate matter exposure by using enrichment analysis.

From the kynurenine metabolic pathway, 22 metabolites were identified and compared between the blood samples of the HG and LG groups. The PCA and PLS-DA plots (Figure 2A,B) showed that the kynurenine metabolites of the HG differed from the LG groups. The two-dimensional PCA plot shows an intersection area and some outliers. Principal components 1 and 2 (PC1 and PC2) accounted for 22.9% and 14.3%, respectively. The two dimension of PLS-DA were used to observe the difference in the kynurenine metabolites between the groups. The PLS-DA plot clearly showed the separation between the groups. Components 1 and 2 revealed 21.4% and 14%, respectively. Xanthurenic acid and tran-3-hydroxykynurenine were highly related to the HG group, but L-tryptophan and melatonin were highly related to the LG group (Figure 2C). Diseases relating to the kynurenine pathway after long-term particulate matter exposure were predicted using enrichment analysis. High exposure to PM could induce cancer in many organs, including the colon, ovary, liver, white blood cells, and stomach. Furthermore, neurodegenerative diseases (Alzheimer’s disease) and kidney diseases (uremia, chronic renal failure) were induced. The data are shown in Figure 3.

### 3.5. The Level of PM2.5 and PM10 in Atmosphere

**Figure 4:**
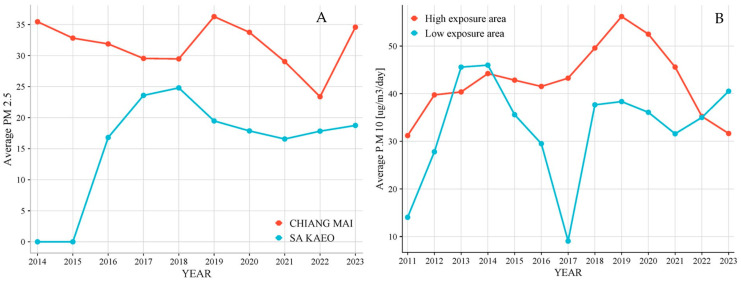
The average levels of PM2.5 (**A**) and PM10 (**B**) represent high-exposure areas (Mae Jam, Chiang Mai) and low-exposure areas (Wata Nanakorn, Sa Kaeo) from June 2014 to December 2023.

The levels of PM2.5 and PM10 were collected from June 2014 to December 2023 by the Thai Metrological Department. The average levels each year at the Mae Jam District (high exposure area) and the Wata Nanakorn District (low-exposure area) were plotted and presented in Figure 4. PM2.5 and PM10 concentrations were higher in areas with high exposure, ranging from 23–36 µg/m^3^/day and 32–59 µg/m^3^/day respectively, compared to low-exposure areas with ranges of 17–25 µg/m^3^/day and 9–45 µg/m^3^/day.

### 3.6. Correlation between Time Spent Living in the Area, PM2.5 and PM10 Concentration, and Kynurenine Metabolites

**Figure 5:**
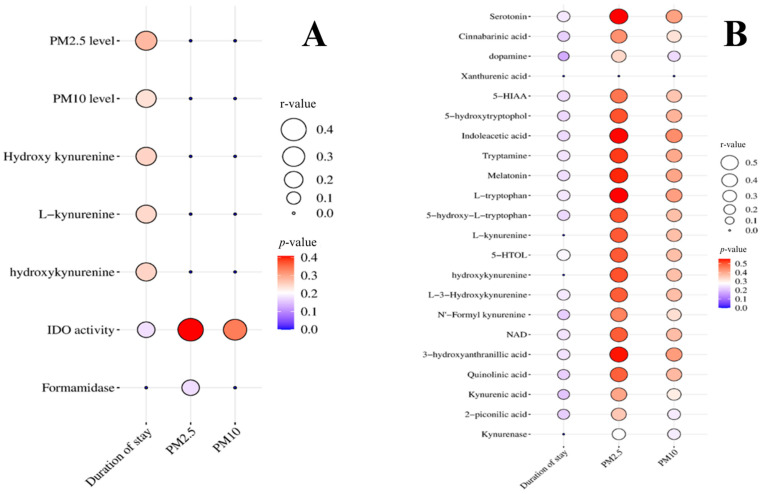
Spearman’s rank correlation coefficients between the time spent living in the area, PM2.5 and PM10 concentration, and kynurenine metabolites when (**A**) and (**B**) present positive and negative correlation, respectively.

The time spent living in the area was significantly positively correlated with the dose of PM2.5 and PM10, as well as the blood levels of hydroxykynurenine, L-kynurenine, IDO, and formamides activity. The daily dose exposure of PM2.5 and PM10 significantly positively correlate to IDO acidity. However, the time spent living in the area and the dose of PM2.5, and PM10 had a negative correlation with the kynurenine metabolites and enzyme activity involved in the kynurenine pathway. Spearman’s rank correlation coefficients between the time spent living in the area, PM2.5 and PM10 concentration, and kynurenine metabolites are presented in Figure 5.

## 4. Discussion

**Figure 6:**
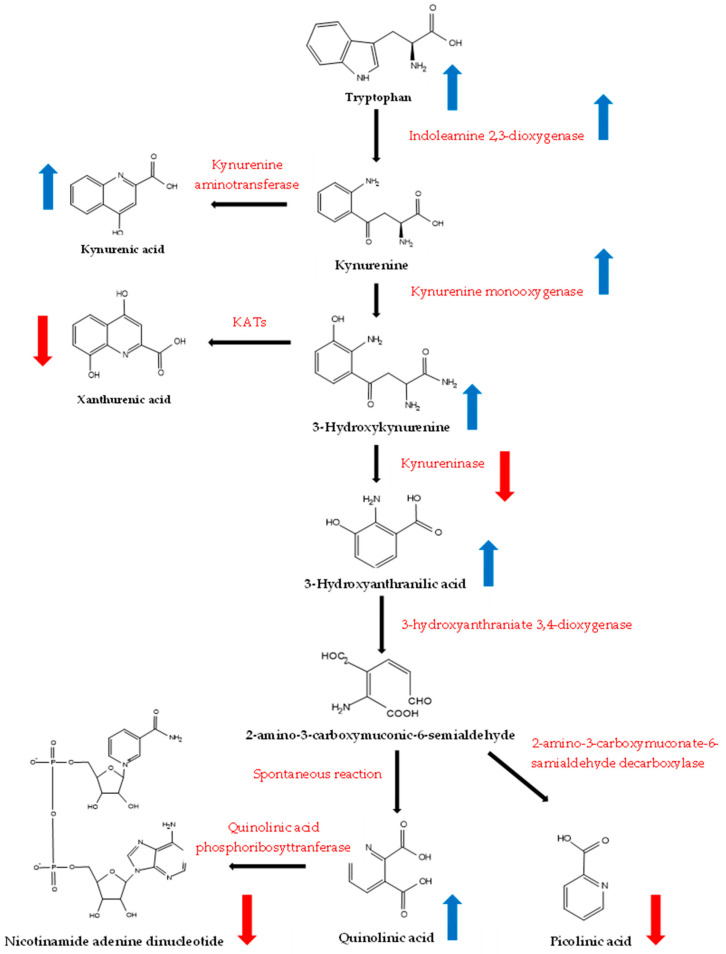
The summarization effect of PM2.5 and PM10 exposure to kynurenine pathway.

Our most important finding is that long-term exposure to PM2.5 and PM10 affected kynurenine metabolism, and the summarized pathway is presented in Figure 6. The air pollution problem in Thailand is associated with fine particulate matter based on a seasonal and differing period in each area [28]. Chiang Mai, the biggest city in northern Thailand which is located on a plain between high mountains, began the period of haze in January and stopped in April. During haze season, Chiang Mai was found to have the top ten dust levels leading to air pollution in the world. Biomass burning from agriculture and wildfires is a major source of PM2.5 and PM10. Sa Kaew is a city in eastern Thailand located on the plain near the sea. Biomass burning from agriculture is a significant source of PM2.5 and PM10, but the level is less than in Chiang Mai. The average value of PM2.5 in the high- and low-exposure areas was 31.7 and 19.5 µg/m^3^/day, respectively. The average levels of PM10 in high- and low-exposure areas were 42.3 and 32.8 µg/m^3^/day, respectively. The high-exposure area exposed PM2.5 and PM10 more than the low-exposure areas, by about 1.6 and 1.3 times. The average levels of PM2.5 and PM10 in both the high- and low-exposure areas in Thailand were higher than the World Health Organization (WHO) air quality guideline level [29].

The demographic data, including sex, age, weight, height, heart rate, and systolic-diastolic pressure, between the groups were similar, and then the factors involved in the kynurenine metabolic pathway were controlled. Age [30] and sex [31] are essential factors in the kynurenine catabolism and need to be controlled.

In this study, the blood samples were collected from healthy volunteers who have lived (more than 15 years) in the high- and low-exposure areas, and the metabolites related to the kynurenine pathway were evacuated. The correlation data revealed that the exposure levels of PM2.5 and PM10 depended on the time spent living in the area and the individual’s weight. Those who stayed for a longer duration in high-exposure areas was exposed to more PM2.5 and PM10 than those who stayed in low-exposure areas. Furthermore, the more time spent living in the area altered the kynurenine pathway by increasing hydroxykynurenine, L-kynurenine, IDO, and formamides activities.

Serotonin, cinnabarinic acid, xanthurenic acid, 5-hydroxytryptophol, indoleacetic acid, tryptamine, melatonin, L-tryptophan, 5-hydroxy-L-tryptophol, indoxyl, 2-aminobenzoic acid, 5-HTOL, hydroxykynurenine, L-3-hydroxykynurenine, *N*-formyl kynurenine, 3-hydroxy anthranilic acid, kynurenic acid, and picolinic acid levels decreased in the participants who had lived in the high-exposure area. The kynurenine pathway can be activated after inducing PM2.5 in a rat model [32]. Up-regulation of xanthurenic acid and kynurenic acid may be induced by activation of tryptophan 2, 3-dioxygenase. The level of kynurenic acid was decreased, and the level of quinolinic acid was increased in BALB/c mice exposed to PM2.5 [33].

The kynurenine pathway is a major tryptophan metabolic pathway that, under physiological conditions, metabolizes tryptophan (>95%) into *N*-formyl kynurenine and an array of downstream neuroactive metabolites [34]. The tryptophan to kynurenine conversion process is enabled by two rate-limiting enzymes: indoleamine 2, 3-dioxygenase and formamidase. Increasing *N*-formyl kynurenine is a primary step for neurotoxic chemicals, including 3-hydroxykynurenine, 3-hydroxyanthranilic acid, and quinolinic acid, while kynurenine, kynurenic acid, and picolinic acid are generally thought to have neuroprotective properties [35]. The compensation or homeostasis balances in the human system might protect the body from degenerative disease activation [36]. Oxidative and antioxidant pathways should be investigated to confirm the homeostasis balance.

The values of *N*-formyl kynurenine/L-tryptophan (IDO) and L-kynurenine/*N*-formyl kynurenine (formamidase) significantly increased, but 2-Aminobenzoic acid/L-kynurenine (kynuredidase) and L-3-hydroxykynurenine/L-kynurenine (kynurenine 3-monooxygenase) significantly decreased in the high-exposure group more than the low-exposure group. IDO and formamidase are key enzymes in the tryptophan catabolism to kynurenine [37]. The functions of IDO and kynurenine are more focused on promoting tumor progression and eliciting tumor-microenvironment immune suppression. Our results showed that long-term exposure to PM activates IDO and formamides activities. A possible mechanism might be induced by oxidative stress or the AHR receptor, which might be proved.

The kynurenine pathway is a metabolic pathway that metabolizes the amino acid tryptophan. In inflammatory conditions, such as those induced by cytokines, the activity of the enzymes involved in the kynurenine pathway can be upregulated. Inflammation can indeed induce the kynurenine pathway [38]. Cytokines are signaling molecules that are released by immune cells in response to infection, injury, or inflammation. Specific cytokines, such as interferon-gamma (IFN-γ), tumor necrosis factor-alpha (TNF-α), and interleukin-1 beta (IL-1β), can stimulate the expression of the enzyme indoleamine 2, 3-dioxygenase (IDO) [39,40].

Activation of the kynurenine pathway by PM2.5 exposure has several implications, including immunomodulation, oxidative stress and neurotoxicity. Metabolites of the Kynurenine pathway, such as kynurenine and its derivatives, can modulate immune responses. Increased production of these metabolites in response to PM2.5 exposure may contribute to immune dysregulation and lung inflammation. Some studies suggest that activation of the kynurenine pathway in response to PM2.5 exposure may lead to generating reactive oxygen species (ROS) and oxidative stress, which can further exacerbate inflammation and tissue damage. Metabolites of the kynurenine pathway, such as quinolinic acid, have neurotoxic properties and may contribute to neuroinflammation and neuronal damage.

Exposure to PM2.5 can indeed lead to activation of the kynurenine pathway. PM2.5 particles, due to their small size, can penetrate deep into the respiratory system upon inhalation, where they can directly interact with lung tissues and trigger inflammatory responses [8]. Previous studies have shown that exposure to PM2.5 is associated with increased levels of pro-inflammatory cytokines such as interleukin-6 (IL-6), tumor necrosis factor-alpha (TNF-α), and interferon-gamma (IFN-γ) in the lungs [41]. These cytokines can stimulate the expression of enzymes involved in the kynurenine pathway, such as indoleamine 2, 3-dioxygenase (IDO), leading to increased tryptophan metabolism along this pathway [8].

The results showed that in the high-PM2.5-exposure area, there might be a prognosis for cancer, especially colorectal, ovarian, liver, stomach cancer, and leukemia. Moreover, Alzheimer’s disease, neurodegenerative diseases, and kidney diseases (uremia, chronic renal failure) progressed. Air pollution has been reported to have a marked impact on public health. In particular, PM2.5 is classified as a serious health hazard and has shown a strong association with cognitive dysfunction [33]. PM2.5 induces inflammation through various mechanisms, including directly irritating the tissues, generating reactive oxygen species (ROS), triggering inflammation, and activating the immune system, leading to the release of pro-inflammatory cytokines and chemokines. PM2.5 can also enter the bloodstream and reach other organs [42], which can induce systemic inflammation.

PM2.5 in the air can deeply penetrate and distribute through the human body. Metabolomics data revealed that PM2.5 could promote colorectal cancer, ovarian cancer, hepatocellular carcinoma, leukemia, and stomach cancer. An overview of the enriched metabolites set showed that colorectal cancer was highly associated with long-term exposure to PM2.5 and PM10. Colorectal cancer is the second deadliest cancer worldwide and is associated with PM2.5. Odd value of the risk, incidence, and mortality of colorectal cancer were 1.19 [95% CI 1.12–1.28],1.18 [95% CI 1.09–1.28] and 1.21 [95% CI 1.09–1.35]), respectively. In Thailand, the odd value of the risk of colorectal cancer associated with PM2.5 was 1.18 [95% CI 1.07–1.29] [43]. Long-term exposure to PM2.5 has been associated with elevated C-reactive protein levels and an activated state of systemic inflammation. It has been demonstrated that exposure to PM2.5 exacerbates alterations in the makeup of intestinal bacteria leading to increased permeability, impaired gut barrier function, inflammatory cell infiltration, and systemic inflammation [44].

Xanthurenic acid and kynurenic acid are associated with chronic kidney disease. PM2.5 can damage kidney function by inducing endothelial dysfunction, activating the renin-angiotensin system, and inducing inflammation, oxidative stress, and apoptosis in the kidney [45]. Moreover, PM2.5 exposure is associated with Alzheimer’s disease, resulting from inflammation and oxidative stress in the brain [46]. Kynurenine pathway and downregulation products are involved in central nervous system diseases, such as depression, epilepsy, schizophrenia, and other diseases. PM2.5 also causes the alteration of neuronal morphology and synaptic changes, increases amyloid-beta and hyperphosphorylated-tau, and induces the enzyme levels involved in the amyloidogenic pathway [47].

The accumulation of kynurenine and its metabolites in the human body can induce oxidative stress, apoptosis, and inflammation. The alteration of enzyme activities involved in the kynurenine pathway has a significant impact on neurological dysfunction, chronic renal disease, and cancer activation [48]. Therefore, people who live in high-exposure areas should protect themselves and reduce PM2.5 and PM10 sources as a first priority.

## 5. Conclusions

Long-term exposure to ambient PM2.5 disrupts the kynurenine pathway by increasing metabolites related to inflammation, the oxidative pathway, and neurotoxicity. More research is needed to fully understand the mechanisms underlying the effects of PM2.5 on the kynurenine pathway. Evidence suggests that exposure to PM2.5 can influence tryptophan metabolism along this pathway, with potential implications for immune function, oxidative stress, and neurological health.

## Figures and Tables

**Table 1:** Demographic data of participants.

Parameters	High-Exposure Group (*n* = 92)	Low-Exposure Group (*n* = 98)	*p*-Value
Sex: male (%)	43 (46.8%)	36 (35.6%)	0.118
Age (years)	46.8 ± 11.3	47.9 ± 12.4	0.555
Weight (kg)	61.0 ± 11.2	65.2 ± 13.9	0.137
Height (cm)	159.1 ± 12.0	161.6 ± 8.6	0.971
Systolic pressure (mmHg)	127.7 ± 17.5	129.1 ± 21.9	0.085
Diastolic pressure (mmHg)	77.7 ± 11.1	81.2 ± 14.0	0.127
Heart rate (bear/min)	77.3 ± 14.1	83.9 ± 14.1	0.998
Time spent living in the area (year)	43 ± 13	34 ± 14	0.0001 *

Data are expressed as the mean ± standard deviation (S.D.) and * significant difference compared between groups using Mann–Witney U test.

**Table 2:** The biomarkers in the kynurenine pathway in low- and high-PM2.5 and PM10-exposure areas.

Metabolites (µM)	High-Exposure Group (*n* = 92)	Low-Exposure Group (*n* = 98)	*p*-Value
Serotonin	20.88 ± 9.28	40.40 ± 31.73	<0.001 *
Cinnabarinic acid	7.59 ± 4.90	12.25 ± 9.91	0.002 *
Xanthurenic acid	12.49 ± 10.65	23.60 ± 16.78	<0.001 *
5-HIAA	14.01 ± 9.31	14.65 ± 11.73	0.651
5-Hydroxytryptophol	9.64 ± 5.57	11.98 ± 9.35	0.402
Indoleacetic acid	15.71 ± 8.95	26.93 ± 19.57	<0.001 *
Tryptomine	11.43 ± 7.12	22.17 ± 14.95	<0.001 *
Melatonin	10.17 ± 6.13	17.28 ± 12.66	<0.001 *
L-tryptophan	7.59 ± 4.31	20.01 ± 14.11	<0.001 *
5-Hydroxy-L-tryptophan	9.70 ± 5.97	18.91 ± 12.51	<0.001 *
Indoxyl	11.24 ± 6.45	34.33 ± 21.35	<0.001 *
2-Aminobenzoic acid	18.96 ± 11.74	24.88 ± 19.50	0.130
L-kynurenine	11.17 ± 6.48	11.88 ± 9.43	0.502
5-HTOL	6.03 ± 3.39	11.13 ± 1053	0.068
Hydroxykynurenine	1.72 ± 0.92	5.53 ± 5.13	<0.001 *
L-3-hydroxykynurenine	3.54 ± 1.83	5.49 ± 4.84	0.012 *
*N*-formyl kynurenine	3.76 ± 2.27	5.42 ± 4.63	0.038 *
NAD	3.84 ± 2.07	2.31 ± 2.08	<0.001 **
3-hydroxy anthranilic acid	1.86 ± 0.93	11.11 ± 9.09	<0.001 *
Quinolinic acid	9.69 ± 5.30	8.81 ± 9.87	0.428
Kynurenic acid	5.48 ± 3.06	6.33 ± 7.34	0.278
Picolinic acid	3.50 ± 1.83	8.86 ± 10.11	<0.01 *
*N*-formyl kynurenine/L-tryptophan	0.53 ± 0.20	0.29 ± 0.12	<0.01 **
L-kynurenine/*N*-formyl kynurenine	3.22 ± 1.44	2.62 ± 2.06	<0.01 **
Kynurenic acid/L-kynurenine	0.58 ± 0.37	0.52 ± 0.39	0.880
2-Aminobenzoic acid/L-kynurenine	1.72 ± 0.45	2.21 ± 0.94	<0.01 *
L-3-hydroxykynurenine/L-kynurenine	0.34 ± 0.11	0.46 ± 0.15	<0.01 **

The results are presented in mean ± standard deviation (S.D.). * The high-exposure group is significantly lower than the low-exposure group, ** the high-exposure group is significantly higher than the low-exposure group between groups when compared with the Mann–Whitney U test (*p* < 0.05). 5-HIAA = 5- Hydroxyindoleacetic acid, 5-HTOL = 5-hydroxytryptophol, NAD = Nicotinamide adenine dinucleotide.

## Data Availability

The original contributions presented in the study are included in the article, further inquiries can be directed to the corresponding author.
